# Comparative genomics of *Mycobacterium avium* subsp. *hominissuis* strains within a group of captive lowland tapirs

**DOI:** 10.1371/journal.pone.0320499

**Published:** 2025-04-01

**Authors:** Hanka Brangsch, Sandra Marcordes, Anne Busch, Michael Weber, Silver A. Wolf, Torsten Semmler, Dirk Höper, Sten Calvelage, Jörg Linde, Stefanie A. Barth

**Affiliations:** 1 Friedrich-Loeffler-Institut - Federal Research Institute for Animal Health (FLI), Institute of Bacterial Infections and Zoonoses, Jena, Germany; 2 Department of Veterinary Medicine, Cologne Zoo, Cologne, Germany; 3 Theoretical Microbial Ecology, Friedrich-Schiller-University Jena, Jena, Germany; 4 Cluster of Excellence Balance of the Microverse, Friedrich-Schiller-University Jena, Jena, Germany; 5 Friedrich-Loeffler-Institut – Federal Research Institute for Animal Health (FLI), Institute of Molecular Pathogenesis, National Reference Laboratory for Bovine Tuberculosis, Jena, Germany; 6 Robert Koch Institute, Genome Competence Centre (MF1), Berlin, Germany; 7 Friedrich-Loeffler-Institut - Federal Research Institute for Animal Health (FLI), Institute of Diagnostic Virology, Greifswald - Isle of Riems, Germany; Zhejiang University, CHINA

## Abstract

Within a group of three captive lowland tapirs (*Tapirus terrestris*) suffering from clinically apparent mycobacteriosis, non-tuberculous *Mycobacterium avium* subsp. *hominissuis* (MAH) strains were isolated from the animals and the tapir’s enclosure. Based on MIRU-VNTR findings, which identified two closely related INMV profiles (124 and 246), a micro-evolutionary event was assumed, and four available MAH strains were submitted to whole genome sequencing (short- and long-read technologies). Surprisingly, the differences based on single nucleotide polymorphisms (SNPs) were exceptionally high between the four strains, i.e., between 841 and 11,166 bases, due to a strong impact of homologous recombination. Thus, an ad hoc core genome multilocus sequence typing (cgMLST) scheme was created and pangenome analysis was conducted for determining the genomic similarity between the strains. The INMV246 isolate obtained from sputum on the enclosure floor and one INMV124 isolate of tapir #2 showed the highest congruence, suggesting that both originated from a shared source. The other two INMV124 isolates were genomically distinct from these strains. Nevertheless, in all four strains two plasmids were detected, which were highly conserved between the strains. The study showed that the genomic variability between MAH strains isolated from the same site within a short period of time can be exceptionally high and the influence of homologous recombination needs to be considered when determining MAH strain relationships, particularly via SNP analyses.

## Introduction

The family of *Mycobacteriaceae* comprises the genus *Mycobacterium*, which is divided into two major groups, members of the *Mycobacterium tuberculosis* complex (MTC) and non-tuberculous mycobacteria (NTM). Among these, *Mycobacterium tuberculosis, M. bovis,* and *M. caprae* represent members of the MTC and are the main causative zoonotic pathogens for tuberculosis in humans and animals alike [1]. However, also NTM can cause mycobacterial infections, which are clinically indistinguishable from tuberculosis. One of the clinically most relevant NTM in humans and animals is *M. avium*. *M. avium* comprises four subspecies: *M. avium* subsp. *avium* (MAA) and *M. avium* subsp. *silvaticum* (MAS), both causing avian tuberculosis primarily in birds, *M. avium* subsp. *paratuberculosis* (MAP), which causes Johne’s disease in several ruminant and livestock species, and *M. avium* subsp. *hominissuis* (MAH) [2]. MAH exhibits the broadest host spectrum of NTM and is capable to cause disease in immune-compromised humans and animals, affecting nearly all organ systems. *M. avium* is part of the *M. avium* complex (MAC), which also includes *M. arosiense, M. bouchedurhonense*, *M. chimaera, M. colombiense*, *M. intracellulare*, *M. lepraemurium*, *M. marseillense*, *M. timonense*, and *M. vulneris* [3,4].

Several studies investigated the genetic relatedness across members of the MAC using MIRU-VNTR, MLST, RFLP or whole genome sequencing (WGS) analyses [2,5–7]. This revealed that MAA and MAS are nearly indistinguishable from another based on their genetic repository, but illustrate clear differences in phenotypic culture behaviour. Likewise, MAP strains are highly similar to another, forming a tight phylogenetic clade including sheep and cattle lineages [8]. In contrast, MAH strains are clearly separated from the MAA/MAS and MAP clusters and their group exhibits increased genomic heterogeneity [2]. In recent years, a higher horizontal gene transfer rate in MAH compared with other mycobacteria was hypothesized based on WGS data [9,10], as at least eight genomic islands were identified within the MAH’s accessory genome, which integrated into the same genomic region. The size of this genomic island and its gene content differed strongly between strains [10].

Recently, we isolated MAH strains as mycobacteriosis causing agents from a group of three lowland tapirs suffering from pulmonary disorder [11] and were able to follow-up the tapirs during treatment, resulting in a complete temporal and clinical resolution of the animals [12]. During this process, we isolated MAH from various sources, including environmental samples (water of the indoor pool, tapirs sleeping bed), as well as from the animals directly (broncho-alveolar lavage) or through animal-associated samples (sputum) [11]. Subsequent characterization of the MAH strains was performed using MIRU-VNTR [2]. Four of the five analysed strains hereby illustrated an identical pattern (INMV124), while a single strain (isolated from a sputum sample from the floor) differed in locus 32, exhibiting only four instead of eight repeats, subsequently forming a new profile INMV246. Although MAH is nearly ubiquitous within the environment [13], we hypothesized that this repeat number difference in the strain may be the result of a micro-evolutionary event, as the presence of only four repeats in locus 32 actually only occurs in two out of 251 INMV profiles [14] and INMV 124 is not frequently described within the current literature [15,16].

To address our hypothesis of a micro-evolutionary event, we performed WGS on four strains, two isolated in 2017 (INMV124 and INMV246) and two in 2018 (INMV124) using both, short- and long-read sequencing technologies. By employing multiple genotyping methods, we compared the strains on per-base- and gene-level, allowing us to describe the high genomic variability between MAH strains originating from the same setting in great detail.

## Materials and methods

In 2017 and 2018, three captive lowland tapirs (*Tapirus terrestris*) with pneumological diseases were subjected to a complex diagnostic procedure in order to identify the underlying pathogen [11]. The animal enclosure was also examined as part of this investigation. In addition to other non-tuberculous mycobacteria, five MAH strains were isolated, two from the environment and three from animal-derived samples. Of these five, four strains were available for the upcoming characterization.

Supplementary information to this manuscript was deposited with Zenodo (https://doi.org/10.5281/zenodo.12918954).

### Strains and DNA preparation

The cryo-conserved MAH strains (Table 1) were inoculated in 7H9 broth supplemented with oleic acid, albumin, dextrose, and catalase (OADC) (Becton Dickinson, Heidelberg, Germany) and incubated (37°C) until bacterial growth was evident. Bacteria were pelleted and resuspended in 500 µl 1x TE buffer. Extraction of the genomic DNA was performed using the cetyltrimethyl-ammonium bromide (CTAB) method with RNAse A digestion as described previously [17].

**Table 1:** MAH strains included in the current study [11].

Strain	Origin	INMV pattern
17MA0524	water sample of the indoor pool	INMV124
17MA0531	tapir’s sputum on the floor	INMV246
18MA0850	broncho-alveolare lavage of tapir #1	INMV124
18MA0854	broncho-alveolare lavage of tapir #2	INMV124

### DNA sequencing

#### Short-read technology.

Whole genome sequencing was performed at the Friedrich-Schiller-Universität (Jena, Germany) for the two strains from 2017 using the Illumina MiSeq platform to generate 150 bp paired-end reads, obtaining a per-base coverage greater than 40X. The two strains from 2018 were sequenced using Illumina NovaSeq 250 bp paired-end sequencing at Eurofins Germany (Koblenz, Germany).

#### Long-read technology.

The long-read sequencing library of the two strains isolated in 2017 was prepared using the Rapid Barcoding Kit SQK-RBK004 (Oxford Nanopore Technologies Ltd, Oxford, United Kingdom) at the Robert Koch Institute (Berlin, Germany). Libraries were run using R9.4.1 flow cells (Oxford Nanopore Technologies Ltd, Oxford, United Kingdom) for 48 hours on a GridION device (Oxford Nanopore Technologies Ltd, Oxford, United Kingdom). At the Friedrich-Loeffler-Institut (Greifswald - Isle of Riems, Germany), the two strains from 2018 were sequenced on a PromethION P2 solo device (Oxford Nanopore Technologies Ltd, Oxford, United Kingdom) using R10.4.1 flow cells. These libraries were prepared with the Rapid Barcoding Kit SQK-RBK114-24 (Oxford Nanopore Technologies Ltd, Oxford, United Kingdom) and run for 72 hours. All raw sequencing data and the assembled genomes have been made publicly available by submission to the European Nucleotide Archive (ENA) under the project number PRJEB77047.

### Post-sequencing processing of raw data

Basecalling of the long-read data was conducted by Guppy v6.0.1 (Oxford Nanopore Technologies Ltd, Oxford, United Kingdom) for the GridION data and Guppy v6.5.7 for the PromethION data, always in super-accuracy mode, i.e., using dna_r9.4.1_450bps_sup and dna_r10.4.1_e8.2_400bps_5khz_sup, respectively. The data quality was assessed with NanoPlot v1.32.1 [18]. Short-read data quality was verified using FastQC v0.11.5 (https://www.bioinformatics.babraham.ac.uk/projects/fastqc/). Kraken2 v2.1.1 [19] was employed for determining species identity and the absence of sample contaminations. The latter was also checked by ConFindr v0.8.1 [20], which detects intraspecific contamination in particular.

### Genome assembly, annotation and comparison

A hybrid assembly approach using Unicycler v0.4.8 [21] in conservative mode was chosen to combine both Illumina and Nanopore reads. Starting positions of all assembled contigs were adjusted by the fixstart function of Circlator v1.5.5 [22]. The assembly graphs were visually inspected for circularity using Bandage v0.8.1 [23]. Assembly quality statistics were determined by QUAST v5.0.2 [24]. The assemblies were subsequently annotated using Bakta v1.7.0 with database version 5.0 [25]. Insertion sequences were detected by ISEScan v1.7.2.3 [26]. Completeness of the assemblies and contamination status was checked using BUSCO v5.7.1 [27]. Complete assemblies were visualized using GenoVi v0.4.3 [28]. Multiple genome alignments were generated with Mauve v2.4.0 [29] employing the progressiveMauve algorithm. Using Platon v1.5.0 [30], the assemblies were further screened for the presence of putative plasmid-borne contigs. A comparison of these plasmid-borne contigs was visualized with BLAST Ring Image Generator v0.95 [31]. For assigning hypothetic products of annotated plasmid genes to Cluster of Orthologous Groups (COGs), plasmid annotation files were analysed with eggnog-mapper v2.1.12 [32]. The results were matched with the classification of NCBI’s Database of Clusters of Orthologous Genes (https://www.ncbi.nlm.nih.gov/research/cog/; accessed 10.01.2024).

Raw assemblies were screened for the presence of virulence-related genes using the VFanalyzer pipeline of the Virulence factor database online (accessed: 12.01.2024) [33]. AMRFinder [34] as well as Abricate v1.0.1 (https://github.com/tseemann/abricate) in conjunction with the databases CARD [35] and Resfinder [36] were used for detecting antimicrobial resistance-associated genes.

### Species identification and allele-based typing

*In silico* PCR was utilized for multiple purposes, employing the perl script in_silico_PCR by Egon Ozer (https://github.com/egonozer/in_silico_pcr). Firstly, species identity was verified using primers by Bannantine, Stabel [37]. Secondly, *in silico* genotyping was conducted, i.e., mycobacterial interspersed repetitive unit (MIRU)-variable number tandem repeat (VNTR) typing using primers by Thibault, Grayon [38]. The sizes of the *in silico* PCR products were compared with the MAC-INMV-SSR Database for allele assignment (http://mac-inmv.tours.inra.fr/) [14]. Additionally, the genes *sodA*, *recF*, *groEL1* and *hsp65* were detected *in silico* [7,39] and corresponding sequences aligned using MAFFT v7 [40] to assess base substitutions. In S6 table all used primers are listed. Alignments were visualized using pyBoxShade (https://github.com/mdbaron42/pyBoxshade).

The average nucleotide identity (ANI) was calculated through fastANI v1.1-h4ef8376_0 [41] and the results were visualised in a heatmap by the ComplexHeatmap package in R [42]. Publicly available MAH genomes (downloaded from RefSeq on 22.02.2023) were included in this analysis (n = 208). Assemblies were subjected to *in silico* Multilocus Sequence Typing (MLST) using mlst v2.23.0 (https://github.com/tseemann/mlst) and the 8-loci scheme mycobacteria_2 [43] from PubMLST [44].

### SNP typing and recombination detection

NCBI’s Short Read Archive (SRA) was browsed for publicly available raw sequence data of MAH (accessed on 18.08.2023), which were downloaded and compared to the tapir-associated strains. In order to exclude contaminations, the raw data were filtered with Kraken2 using a cut-off of 90% reads assigned to the *Mycobacterium avium* complex. Samples that fell below this threshold were verified for the type of contamination. If contamination was caused by non-bacterial reads (i.e., human or viral DNA), the contamination was not considered disruptive for further analysis. The samples were then assembled using Shovill v1.0.4 (https://github.com/tseemann/shovill), checked with QUAST and the MLST sequence type was determined as described above. Assemblies that ranged in size from 5 to 5.5 Mb, had a GC content of approximately 69.2% and comprised a maximum of 300 contigs were considered for further analysis.

Snippy v4.6.0 (https://github.com/tseemann/snippy) was used for core genome single nucleotide polymorphism (cgSNP) typing, employing the hybrid assembly of 17MA0531 as reference genome. The number of pairwise cgSNPs was calculated using snp-dists v0.7.0 (https://github.com/tseemann/snp-dists) and the alignment was used as input for SplitsTree4 v4.18.1 [45] with default settings (chartransform=Uncorrected_P; disttransform=NeighborNet; splitstransform=EqualAngle) for calculation of a phylogenetic network by the split decomposition method. SplitsTree’s implemented pairwise homoplasy index (PHI) test was used for the detection of genomic recombination events. Subsequently, outliers were removed from the analysis and the cgSNP alignment was filtered for recombination events using Gubbins v3.2.1 [46] as described in the Snippy manual. Based on the outlier- and the recombination-adjusted core genome SNP alignments, two maximum likelihood trees were calculated by RAxML v8.2.12 [47] with the GTRCAT model, the -V option and Lewis ascertainment bias correction as recommended in the RAxML manual. Trees were visualised with FigTree v1.4.3 (http://tree.bio.ed.ac.uk/software/figtree/). Recombination sites were visualized using the gubbins_draw.py script as implemented in Gubbins. Furthermore, the r/m (base substitutions caused by recombination/point mutations) and rho/theta (number of recombination events/point mutations) ratios were calculated for the branches by Gubbins.

### cgMLST analysis

For creating an *ad hoc* core genome multilocus sequence typing (cgMLST) scheme, all assemblies (n = 208) from NCBI RefSeq were downloaded (accessed on 22.02.2023), that were either denominated *M. avium*, *M. avium* subsp. *avium* or *M. avium* subsp. *hominissuis*. The species identity was verified through *in silico* PCR using primers from Bannantine, Stabel [37]. Assemblies with an MAH-specific amplicon were analysed using QUAST and fastANI. Assemblies that met the following criteria were included as query genomes: high similarity (ANI ≥ 99% to at least one of the four studied isolates), high quality (max. 2 Ns per 100,000 bp), high continuity (max. 200 contigs). For determination of suitable target genes, *M. avium* strain 104 (GCF_000014985.1) was used as seed genome. The cgMLST scheme was generated by the MLST + target definer incorporated in Ridom SeqSphere + v7.7 [48] with default parameters. Subsequently, the scheme was used for the assessment of allelic differences by minimum spanning and neighbour joining methods, both implemented in SeqSphere + . This neighbour joining tree was compared to both maximum likelihood trees from the cgSNP analysis by i) visualizing leaf position differences with a tanglegram generated by Dendroscore v3.5.9 [49] and ii) calculating the Robinson-Foulds symmetric distance and percentage of edge similarity using the ETE Toolkit v3.1.3 [50]. Analogously, both cgSNP-based trees were subsequently compared.

### Pangenome analysis

Based on the MLST sequence type, a set of foreign data was chosen for pangenome analysis including the new MAH isolates. This set included the RefSeq and Genbank assemblies used for ANI and cgMLST as well as the assembled Illumina-only data from SRA. In order to ensure reliable results, assemblies should have max. 200 contigs and an N50 value of at least 100,000 bp to be included in the analysis, as determined by QUAST. The assemblies were annotated using Bakta and the pangenome was determined by Panaroo v1.3.2 [51]. Based on the core gene alignment, a maximum likelihood tree was calculated using RAxML with the GAMMA model of rate heterogeneity. This tree together with the gene presence-absence matrix generated by Panaroo were visualized by Phandango [52]. Predicted genes that were unique to single strains were extracted from the gene_presence_absence table and assigned to Cluster of Orthologous Groups (COGs) of proteins using COGclassifier v1.0.5 (https://github.com/moshi4/COGclassifier). The BLASTn online tool [53] was used for finding the closest matches with other genomic sequences.

## Results

### Genome characterisation

#### Chromosome.

This study aimed at investigating the genetic relationship of four MAH isolates retrieved from a single enclosure in a zoo. By employing a hybrid assembly approach using the combined sequencing depth of both Illumina and Nanopore data, complete, closed chromosomes could be obtained for three strains, resulting in genomic sizes of about 5.15 Mb (Table 2). Solely for strain 17MA0524 the chromosome remained fragmented and approximately 150,000 bp larger compared to those of the other strains. This strain showed the lowest median read length as well as the lowest read quality of Nanopore data (S5 table). However, the GC content, as well as the number of annotated coding sequences (CDSs) was similar to that of the other three strains, i.e., approx. 69.2% GC and 4,757-4,772 annotated genes. No contaminating reads were detected for any of the strains, as determined by ConFindr that screens the short reads. Also, BUSCO, that checks for presence of universal single-copy orthologous genes in assemblies, did not find duplicates that would indicate contamination (S5 table). Thus, the strains were considered to be free of contamination.

**Table 2:** Results of genome assembly using a combination of long and short reads.

Strain	Component	Contigs	Length	N50	Longest segment	Assembly status	GC (%)	CDS
17MA0524	chromosome	84	5,307,785	378,674	1,071,013	incomplete	69.1	4,757
	plasmid pMA01	1	47,679	47,679	47,679	complete	66.4	55
	plasmid pMA02	1	21,658	21,658	21,658	complete	65.4	31
17MA0531	chromosome	1	5,148,629	5,148,629	5,148,629	complete	69.2	4,772
	plasmid pMA01	1	46,253	46,253	46,253	complete	66.4	53
	plasmid pMA02	1	20,233	20,233	20,233	complete	65.4	27
18MA0850	chromosome	1	5,160,223	5,160,223	5,160,223	complete	69.2	4,772
	plasmid pMA01	1	51,700	51,700	51,700	complete	66.2	55
	plasmid pMA02	1	20,233	20,233	20,233	complete	65.4	27
18MA0854	chromosome	1	5,132,125	5,132,125	5,132,125	complete	69.2	4,762
	plasmid pMA01	1	46,253	46,253	46,253	complete	66.4	53
	plasmid pMA02	1	20,233	20,233	20,233	complete	65.4	27

**Fig 1:**
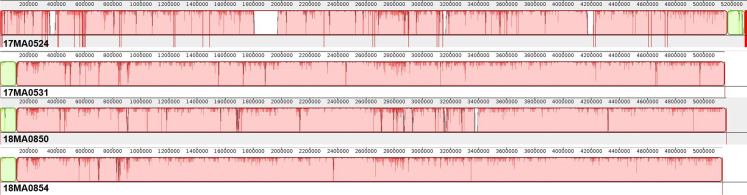
Alignment of chromosome contigs of four MAH strains from Germany. Identical colours show similar genome blocks, whereas blank spaces indicate the presence of strain-specific genome segments. Red vertical lines in assembly of 17MA0524 denote contig boundaries.

By aligning the chromosomal sequences, it was found that they were also highly similar in their genomic structure (Fig 1). Particularly, 17MA0531 and 18MA0854 showed high congruence, whereas in the other two isolates, strain-specific segments were identified, which were not present in any of the other three strains. Particularly, a ~ 170 kb large segment was remarkably only present in 17MA0524. Furthermore, only a single segment of a contig in this strain, indicated in green in Fig 1, was located at a different genomic position.

The genomes were screened for the presence of antimicrobial resistance- (AMR-) and virulence-associated genes. The only hits for AMR determinants (n = 4) were obtained from the database CARD with identical results for all four strains (S1 Table). These represented chromosomally encoded, common actinobacterial genes: A variant of *rpoB* (*rpoB2*), encoding the subunit of the RNA polymerase, as well as *rbpA*, coding for an RNA polymerase-binding protein, and the *M. tuberculosis* transporter-encoding *efpA* were detected and predicted to confer resistance to rifamycin. Additionally, a transcriptional activator was found that illustrated similarity to MtrA, which controls a multidrug efflux pump in *Neisseria* sp., thereby conferring resistance to macrolides and penams.

Overall, 212 different genes coding for proteins involved in virulence were identified (S2 Table), some of which were encoded multiple times on the genomes. The results were largely in agreement with virulence determinants of MAH strain 104. Among the detected genes there were the mammalian cell entry operons *mce1-5*, *mce7*, *mce9*, secretion system genes *esx2-5* and mycobactin synthesis genes. The sets of detected genes were mostly identical between the four strains. In 17MA0524, no *fbpB*, encoding a part of the antigen 85 complex, was found and fewer copies of PPE4 (n = 6) than in the other strains (n = 8-9) were detected. Conversely, 18MA0850 showed the highest number of PPE4 copies (n = 9) as well as two additional genes involved in phthiocerol dimycocerosate and phenolic glycolipid biosynthesis and transport.

Numerous insertion sequence (IS) elements have been detected in all four chromosomes (S3 Table), i.e., between 59 and 80 elements of at least 15 families. Interestingly, 18MA0854 harboured the least number of IS elements, whereas 18MA0850, which was also isolated in 2018, had the most IS elements and the highest number of elements from different IS families. In general, the most dominant elements were of families IS*1634* (n = 12-19) and IS*256* (n = 22-28).

#### Plasmid characterization.

In the assemblies of all four investigated strains, two smaller, complete and closed contigs were found by inspection of the assembly graphs. These were of similar or even identical in size. They were confirmed to be plasmids by to the detection of genetic determinants for mobilization and conjugation and were therefore termed pMA01 and pMA02. There was a marked difference in GC content between the chromosomes and the plasmids. While for pMA01 the %GC was approximately 3% lower than that of the chromosomes, the difference was even higher for pMA02 ( ~ 4% lower %GC) (Table 2).

The plasmids were analysed for their gene content. Predicted proteins were assigned to COGs when possible.

**Fig 2:**
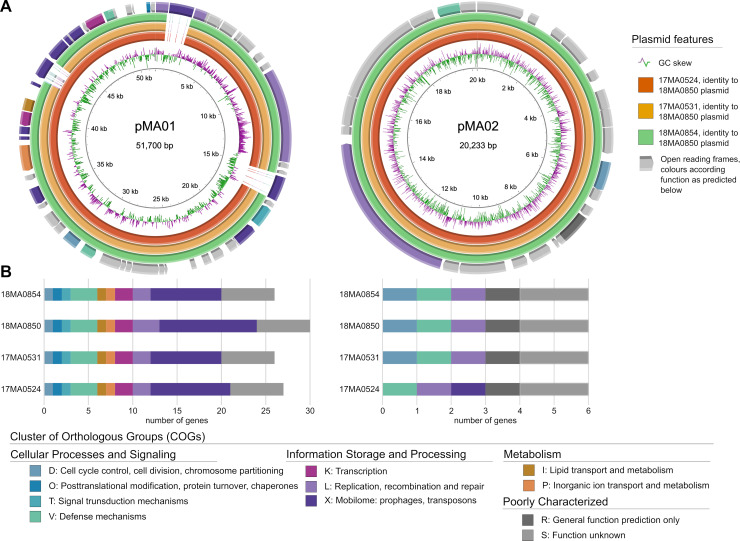
Inter-strain homology of the two plasmids pMA01 and pMA02 based on comparison of the (A) DNA sequences and (B) the content of COGs. (A): plasmids of 18MA0850 served as backbone for aligning corresponding plasmids of 17MA0524 (orange), 17MA0531 (yellow) and 18MA0854 (green). The maps further show the GC skew (second inner ring) and predicted coding sequences coloured accorded to COG. In the lower panel (B) the number of CDS per predicted COG of each plasmid is shown.

The size of plasmid pMA01 varied between the strains, ranging from 46,253 bp in 17MA0531 and 18MA0854 up to 51,700 bp in 18MA0850. In 17MA0524, this plasmid was slightly larger than in the former two strains (47,679 bp) (Table 2). When aligning pMA01 from all strains, it was found that the plasmids were identical in sequence and structure. The increased size in 17MA0524 and 18MA0850 resulted from the insertion of one and three transposon elements, respectively (Fig 2). Overall, seven insertion sequence elements were identified which all pMA01 had in common. Regarding the pMA01 parts present in all strains, the plasmid sequence was highly conserved, differing in maximally two bases between the strains.

Further, several components required for plasmid maintenance and transfer were predicted, e.g., DNA primase and TrwC relaxase proteins for plasmid replication and conjugative transfer. One ORF encoded the ParE toxin of the type II toxin-antitoxin system. The corresponding antitoxin, which was predicted to harbour a PhdYeFM_antitox domain, was encoded upstream. Upstream of these ORFs, but in opposed orientation, a potential transcriptional regulator of XRE-family could be identified. Additionally, another antitoxin family encoding gene was annotated, *brnA*.

Some of the predicted proteins encoded on pMA01 could be involved in stress response and virulence. According to the COG classification, one protein displayed the type VII secretion system ESX-1 protein EspC domain (Pfam: T7SS_ESX_EspC), a mycobacterial virulence-associated factor. In contradiction to that, the protein was annotated as CsbD family protein, involved in stress response, by Bakta. In line with the COG classification, there was an ORF annotated that encoded an EspA-EspE domain-containing protein, which are ESX-1 secretion-associated. Furthermore, a protein homologous to the biofilm regulator BssS was predicted as well as two PPE family proteins.

A BLAST search with the nucleotide sequence of pMA01 revealed high similarity to plasmid pMA100 of MAH strain 88Br (KR997898.1) of unknown origin with a query coverage of 80% and identity of 95.6%.

The size of plasmid pMA02 was identical in all strains (20,233 bp), except for 17MA0524, in which pMA02 was enlarged by 1,400 bp. As with pMA01, this was caused by the insertion of a mutator transposase-like element. In contrast to pMA01, only a single transposon element was annotated in all four pMA02 derivatives, which was from the IS110 family. Further, genes for plasmid maintenance and transfer were identified as well, i.e. *trwC* and *mbcAB*. The latter encoded the mycobactericidal antitoxin MbcA and a RES-domain containing protein (COG5654), which is a group of predicted toxin components of a toxin-antitoxin system. In contrast to pMA01 and pMA02 of the other strains, no *parA* gene was present in pMA02 of strain 17MA0524. Besides that, the plasmid sequence was identical in all strains, except for strain 17MA0524, which exhibited a difference of three bases.

Further, proteins potentially involved in virulence and pathogenicity were predicted. One ORF encoded the transcriptional factor WhiB, an actinobacterial protein that frequently plays a role in virulence and antibiotic resistance. A TipAS-domain containing protein could be involved in antibiotic recognition. The COG classification also predicted an encoded protein of Pfam T7SS_ESX_EspC, a potential virulence factor, located on pMA02.

No significant similarities could be found for the pMA02 sequence in a BLAST search, as the query coverage was maximally 43%. However, the best 14 hits were all plasmids from different MAH strains, all isolated from humans in Japan or the USA.

### Genotyping

#### Allele-based typing.

Eight-loci MLST classified all four strains as sequence type (ST) 9. In the *in silico* MIRU-VNTR analysis, strain 17MA0531 (profile: INMV246) differed in one locus from the other three strains, which were all classified as INMV124. Hence, the data of a previous study [11] was confirmed. Furthermore, nucleotide differences in the housekeeping genes *recF*, *hsp65*, *groEL1* and *sodA* were investigated. While all four strains exhibited identical sequences for the latter two genes, differences could be found in *recF* (S1 Fig): 13 nucleotide substitutions were present in 17MA0524 in this gene, which differentiated this strain from the other three that showed identical sequences. For *hsp65*, no *in silico* PCR product could be obtained for strain 17MA0524 as the primer binding sites were located on different contigs. However, 17MA0531, 18MA0850 and 18MA0854 were identical in *hsp65* sequence.

#### Average nucleotide identity (ANI).

**Fig 3:**
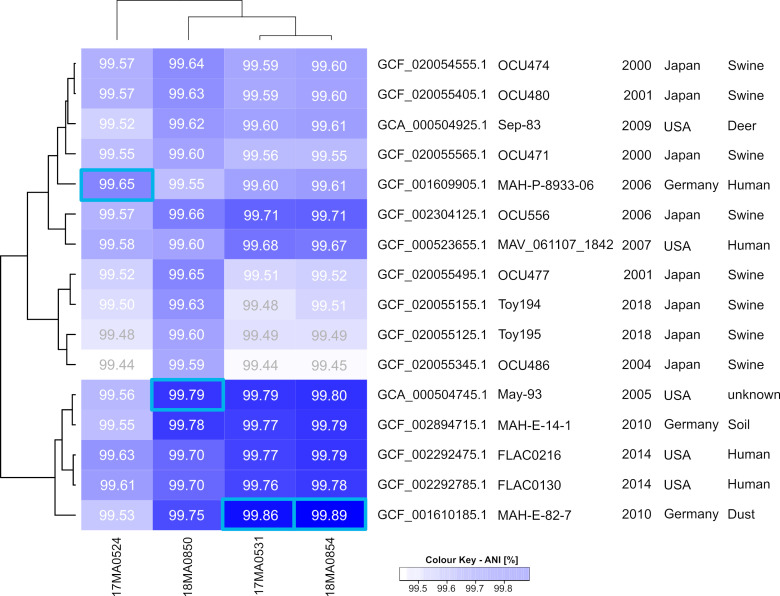
Heatmap of an hierarchical cluster analysis based on average nucleotide identity values between the four tapir-associated isolates and foreign strains. Included were strains that exhibited at least 99.5% ANI to one of the MAH strains of the current study. Blue squares highlight the highest congruence matches.

For calculating the average nucleotide identity between strains, complete genome sequences are compared in pair-wise mode and the average agreement is determined. The ANI to 208 MAH strains retrieved from NCBI was at least 98.3% for the tested strains. Fig 3 depicts the similarities to those isolates which exhibited an ANI of at least 95% to one of the tapir-associated isolates. Particularly, 18MA0854 showed 99.89% identity to a strain isolated from dust in Germany in 2010. Also 17MA0531 showed the highest congruence to this isolate (99.86%). The ANI between 18MA0850 and this German strain was slightly lower (99.75%) than to a strain isolated in the USA from 2006 (99.79%), to which also the other two aforementioned strains exhibited high ANI values. The nucleotide congruence to other strains was lower for 17MA0524, with 99.65% being the highest ANI to a German human strain isolated in 2006, which was comparable to the ANI of a North-American strain also isolated from human in 2014 (99.63% ANI). Thus, 17MA0524 was more similar to strains from a different cluster in the ANI analysis (Fig 3) than the other three investigated isolates of the presented study.

#### cgSNP typing.

**Fig 4:**
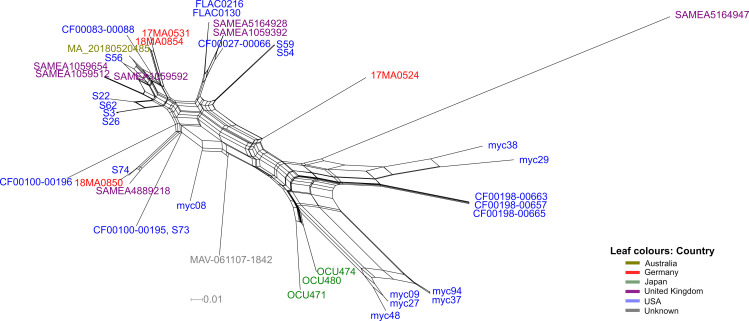
Unrooted phylogenetic network, calculated by split decomposition method based on cgSNP alignment of MAH strains of MLST ST9. The presence of non-linear connections indicates conflicting phylogenetic signals. Strains are coloured according to their place of isolation. The bar indicates base substitution per site.

A core genome SNP approach was chosen for determining the similarity to global MAH strains in detail. In this approach, base differences in genomic regions present in all strains (core genome) are determined, allowing a detailed differentiation of strains. In this analysis, all publicly available Illumina read data sets of strains of MLST ST9 were included, that had passed the contamination check (n = 36 strains). The majority of these strains had been isolated in the USA. The size of the core-genome SNP alignment was 40,149 nucleotides and strains exhibited differences between 0 and > 21,000 SNPs. No cgSNPs were detected on the plasmids of the reference genome 17MA0531, pMA01 and pMA02. This was attributed to the lack of these plasmids in some of the strains of MLST ST9, as will be shown below. The subsequent phylogenetic network analysis (Fig 4) did not yield a distinct clustering of strains with regard to their origin, except for three isolates from Japan that clustered together (OCU471, OCU480, OCU474).

Strain SAMEA5164947 exhibited particularly high SNP differences compared to the other strains in this analysis, with at least 19,122 differing SNPs. Remarkably, the cgSNP differences between the German isolates was high. 17MA0531 and 18MA0854, the isolates from sputum and tapir #2, respectively, shared the highest similarity, but still exhibited 841 cgSNPs. Strain 17MA0524 showed the highest differences to the other three strains (>9,300 cgSNPs). The phylogenetic network analysis placed isolate 18MA0850 near human isolates from the United Kingdom (SAMEA4889218) and USA (S74), whereas no close connection to other strains was observed for 17MA0524. The surprisingly high number of SNP differences between the German genomes as well as the presence of numerous reticulations in the phylogenetic network led to the assumption that the analysis might be influenced by recombination events. Thus, a PHI test for recombination was conducted. The calculated expected mean PHI was 0.523 with an expected variance PHI of 7.48x10^-8^. However, the observed PHI value was 0.228 with a P-value below 0.001 (p < 0.001), indicating a statistically significant evidence for recombination.

Subsequently, the cgSNP analysis was repeated, excluding the outlier strain SAMEA5164947 in order to prevent false-positive recombination signals. This led to a reduction of the core-genome SNP alignment to 32,896 nucleotides. However, the differences between the German strains remained similar (Table 3). To rule out the possibility that mycobacterial PE/PPE sites or insertion elements caused false-positive SNPs, the locations of these sites in the reference genome were masked and the cgSNP analysis repeated. However, excluding these sites did not significantly change the number of SNPs: while exclusion of 57 PE/PPE regions reduced the cgSNP number by 216, excluding the insertion sequence sites merely removed a single position.

**Table 3:** Numbers of detected cgSNP differences between German isolates before removal of outlier SAMEA5164947 (original), after outlier removal (outlier removed) and after filtering for recombination sites (recombination-adjusted).

	Original				Outlier removed				Recombination-adjusted			
Strain	17MA0524	17MA0531	18MA0850	18MA0854	17MA0524	17MA0531	18MA0850	18MA0854	17MA0524	17MA0531	18MA0850	18MA0854
**17MA0524**	0	9,347	11,166	9,302	0	9,455	11,276	9,412	0	35	41	36
**17MA0531**	9,347	0	6,277	841	9,455	0	6,319	847	35	0	20	3
**18MA0850**	11,166	6,277	0	5,493	11,276	6,319	0	5,533	41	20	0	21
**18MA0854**	9,302	841	5,493	0	9,412	847	5,533	0	36	3	21	0

Thus, the core genome SNP alignment was filtered for recombination events. This reduced the 32,896 cgSNPs by 99.1% to 292 base position, indicating that the vast majority of SNP positions was in fact impacted by recombination. The majority of recombination events occurred at internal nodes with a mean ratio of recombination vs. mutation events (r/m) of 17.5, i.e., the number of SNPs located in recombination sites was 17 times higher than outside recombinational sites. Nodes that were not affected by recombination contained highly similar or identical strains. The mean rho/theta ratio for internal nodes, which gives the rate of occurrence of recombination events relative to mutation events, was 0.5. This indicated that mutation occurred twice as often compared to recombination. However, a high proportion of mutational sites were located within recombination blocks, hence the high r/m value.

**Fig 5:**
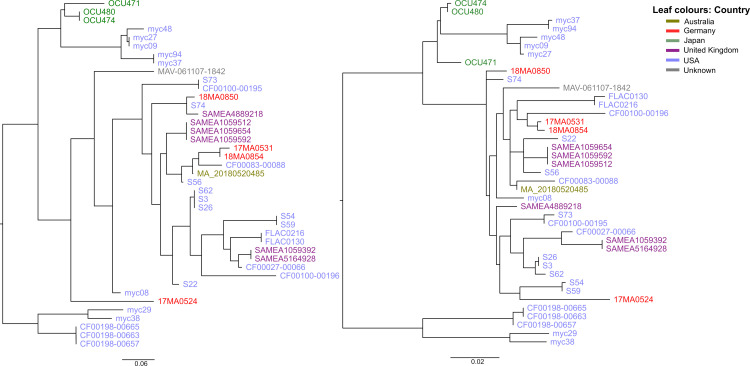
Maximum likelihood trees based on cgSNP alignments of MAH strains of MLST ST9 before (left) and after (right) filtering for recombination sites with Gubbins. Strains are coloured according to their origin country of isolation. The bars indicate nucleotide substitutions per site, illustrating the drastic reduction in SNP differences despite the fact that the branches appear longer.

Interestingly, the relative distances between our strains remained similar. Strains 17MA0531 and 18MA0854 were most identical, now differing in only three SNPs, whereas 17MA0524 showed the highest differences (Table 3). Recombination filtering also changed positioning of few branches in the phylogenetic tree (Fig 5). Particularly, the cluster containing 18MA0850 was dispersed, while 17MA0524 was located at the same branch as two human isolates from the USA.

#### cgMLST.

Allele-based typing methods are considered less sensitive towards genome re-arrangements, because multiple SNPs can be condensed to a single allelic change. Thus, an *ad hoc* cgMLST scheme was created for MAH. Of the 208 assemblies initially downloaded, 192 were identified as MAH and considered for further analysis. After taxonomic and quality filtering 42 assemblies from strains isolated in Japan, USA and Germany (S4 Table) were identified as suitable query genomes. The initial check for taxonomic outliers showed that 83-98.8% of the seed genes could be found in all assemblies. Whereas three genomes with less than 85% of seed genome genes were excluded from the analysis. Thus, 39 assemblies were finally used as query genomes. Using the cgMLST target definer, 3,221 genes (62.5% of the seed genome) were identified as cgMLST targets and 1,586 genes (30.8%) as accessory targets.

**Fig 6:**
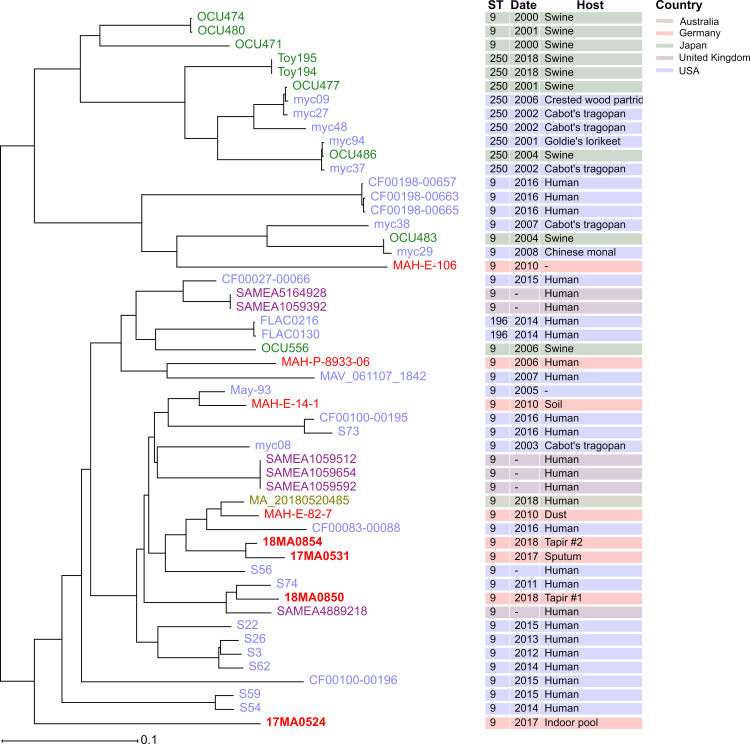
Unrooted neighbour joining tree based on cgMLST allele differences between strains of cgMLST cluster 1 (see S2 Fig). Strains are coloured according to their place of isolation. The MLST ST, date of isolation and host are given at the right side. The bar indicates allelic differences.

This *ad hoc* cgMLST scheme was then used for comparing the strains from the current study with those which were identified as closely related in the traditional MLST, SNP and ANI analyses (76 strains in total). In three assemblies, less than 90% of the cgMLST targets could be detected and they had to be excluded. Two of these were highly fragmented (>1,000 contigs), whereas the third contained a high content of uncalled bases (>2,200 Ns/100 kb). The remaining 73 assemblies exhibited on average 98.4% identified targets (median: 99.2%), which allows reliable analysis. Between 0 and 2,701 alleles difference were observed between the strains. The subsequent minimum spanning tree analysis revealed that strains belonging to the same traditional MLST ST did not necessarily cluster together, or, conversely, different MLST ST’s were place in the same cluster (S2 Fig). In order to take a more detailed look at the similarities between our and the NCBI strains, a cluster threshold of 1,150 alleles was selected, as this included all MLST ST9 strains. Isolates falling in the same cluster as the four MAH strains of the current study (altogether 42 strains) were subjected to neighbour joining (NJ) analysis (Fig 6).

Regarding the MAH strains from this study, the smallest difference was between 17MA0531 and 18MA0854 (115 alleles), while 17MA0524 showed the largest deviation from the other three strains (883-1,122 alleles). Differences to foreign strains were lowest for 18MA0850 compared to a strain isolated in the USA from 2011 (172 alleles). The isolates 17MA0531 and 18MA0854 exhibited 402 and 403 alleles differences, respectively, to a German dust isolate from 2010 (MAH-E-82-7). The allelic profile of 17MA0524 showed the lowest concordance with that of other strains (>877 alleles difference).

The NJ analysis did not show a distinct clustering according to geographic origin, although isolates from Japan were dominant at one branch. Strains of the traditional MLST ST196 and ST250 were not clearly separated from other strains in this cluster, which was dominated by ST9 strains.

Remarkably, the results of the cgMLST NJ analysis regarding the relative positioning of the isolates, were more similar to the SNP typing results before filtering for recombination, as shown in the tanglegram (S3 Fig). Consistenly, the Robinson-Foulds symmetric distance, which is a measure of the node placement difference between two trees, was lowest (26) and the edge similarity highest (83%) between these two trees. The similarity between the cgSNP-based trees before and after recombination filtering was lowest for the considered trees.

#### Pangenome analysis.

The pangenome approach was chosen for assessing and comparing the genetic diversity of the strains on a gene-level. Here, the presence or absence of genes is analysed. Pangenome analysis requires contiguous assemblies in order to avoid false signals from truncated gene sequences. Thus, the available assemblies were screened and 24 assemblies, either deposited at NCBI or generated from SRA data, were selected based on quality and MLST ST, representing 20 different MAH MLST ST9 strains. For four strains both, NCBI assemblies and SRA-based assemblies, were included to control for potential bias from the assembly method. As the cgMLST analysis had shown that MLST ST250 and ST196 were similar to ST9, also assemblies of two ST196 and four ST250 strains meeting the quality requirements were included in the pangenome analysis. All in all, the pangenome was generated based on 26 assemblies from NCBI strains and the four MAH strains under investigation.

A total of 5,896 genes were detected among these strains. The core genome consisted of 4,243 genes (72.0%), which could be detected in at least 99% of strains. About 4.5% (n = 265) of the genes belonged to the soft core genome, i.e., were present in 95-99% of strains. Genes detected in 15-95% of the genomes (shell genes) or in less than 15% of the strains (cloud genes) accounted for 9.5% (n = 563) and 14.0% (n = 825) of the detected genes, respectively.

**Fig 7:**
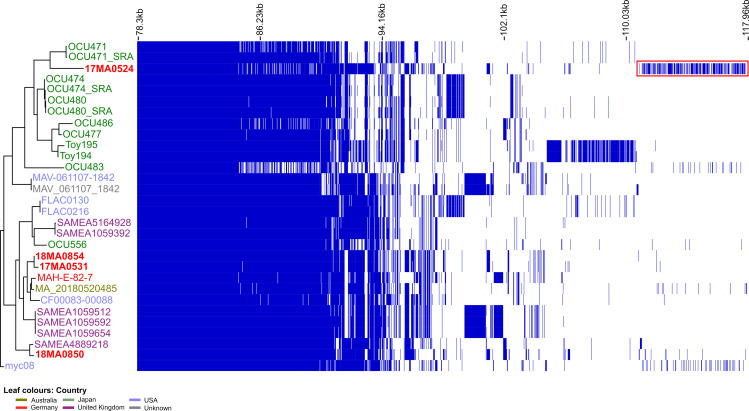
Pangenome analysis of selected MLST ST9, ST250 and ST196 strains with maximum likelihood tree based on core gene alignment. The majority of core genes (78.3 kb) is not displayed for better visibility of differing genes. The red square indicates genes unique to MAH strain 17MA0524. A complete visualisation can be found in S4 Fig.

The phylogenetic analysis based on the core gene alignment (Figs 7 and S4) again showed a high similarity between 17MA0531 and 18MA0854, whereas 17MA0524 was located at a different branch, together with strains isolated from Japan. In agreement with cgMLST analysis, the former two strains clustered with two isolates from human and a dust isolate from Germany. Strain 18MA0850 again showed high similarity to a human isolate from the UK.

The potential impact of the assembly method or pre- or post-assembly filtering steps on the pangenome analysis became apparent for OCU471: several genes could not be found in the SPAdes assembly downloaded from NCBI, but were present in the Shovill assembly generated from SRA data (Fig 7).

**Fig 8:**
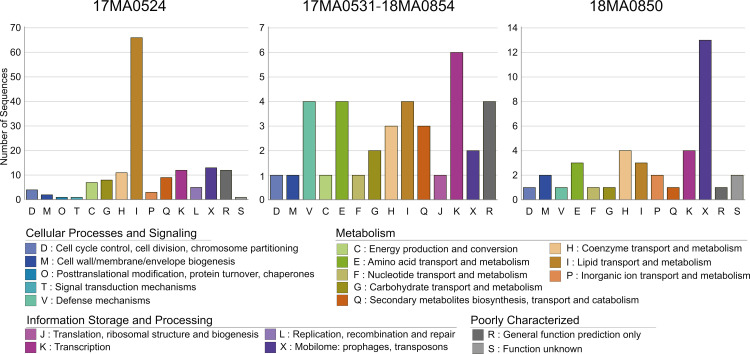
Number of detected genes which could be assigned to COGs that were unique to either one of the tapir-associated isolates 17MA0524 and 18MA0850 or shared by 17MA0531 and 18MA0854 but missing in the other two investigated strains.

Altogether 242 genes were detected that were exclusively found in one of the four strains from the present study, particularly in strain 17MA0524. The visualization of the pangenome showed a block in the 17MA0524 lane representing genes which were exclusively present in this strain (red square). This gene set distinguished 17MA0524 not only from our other strains, but also from all other assemblies of ST9, ST196 and ST250 examined here. From the 225 unique genes identified in the 17MA0524, 155 predicted products could be assigned to COGs. The majority of these were assigned to COGs involved in metabolism and many of these were assigned to COGs of lipid transport and metabolism proteins (n = 66) (Fig 8).

When comparing only the four strains investigated in the present study, 265 CDSs were found in 17MA0524 which were absent in the other three strains. The majority of the genes unique to 17MA0524 were arranged in three contiguous chromosomal fragments of 25,874 bp, 39,839 bp and 161,594 bp, which were also visible in the chromosome alignment (Fig 1). The longest and the shortest of these elements were flanked by transposases of the IS*256* family (IS*666* and IS*1245*) and transposases of the IS*256* and IS1*634* families, respectively. The ~ 40 kb fragment contained a gene encoding a site-specific recombinase of the phage integrase family. The ~ 162 kb and ~ 40 kb fragments comprised the majority of the 225 genes unique to this strain, also compared to the foreign strains in the analysis. Thus, a BLASTN search was conducted, which concluded that these fragments showed the highest identity (100% identity at 99% query coverage) with the chromosome sequence of MAH strain 104, a strain of MLST ST4, isolated from human in 1983.

Strains 17MA0531 and 18MA0854 were almost identical in the pangenome analysis, with only two CDSs unique to one of the two strains, respectively. However, 18MA0854 lacked several transposases of families IS*1634*, IS*110*, IS*1380*. These highly identical strains also shared genes missing in the other two strains studied here (n = 51).

Lastly, strain 18MA0850 harboured 52 unique genes, which were arranged in several smaller fragments (max. 25,517 bp), which were partially associated with IS elements, e.g., IS*256* family elements. Further, several transposon elements were unique to this strain, in agreement with the analysis above.

**Fig 9:**
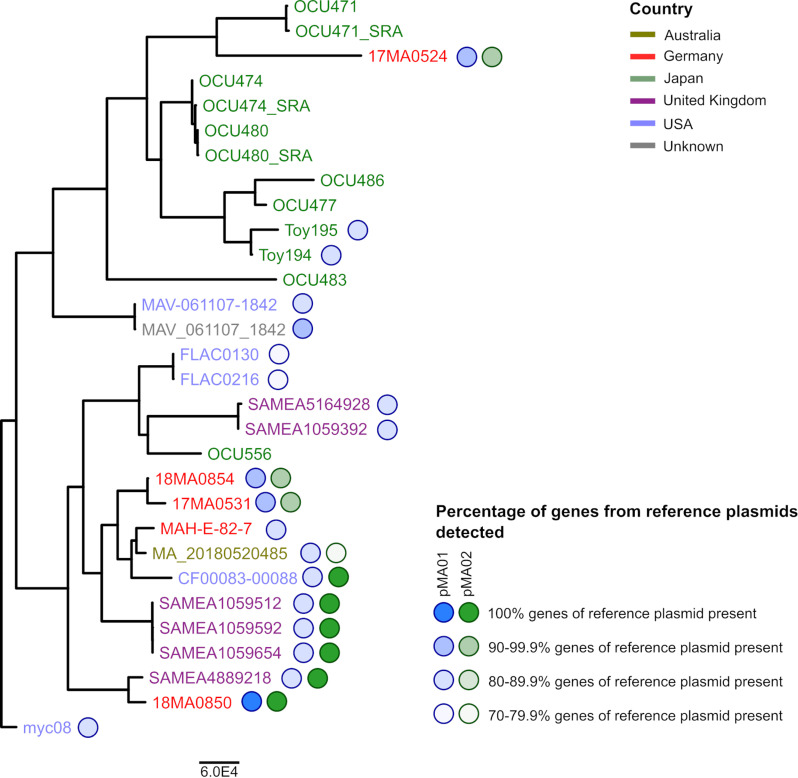
Maximum likelihood tree based on core gene alignment as given in Fig 7, with indicators for the presence of plasmid-borne genes detected in pangenome analysis. Blue circles indicate presence of pMA01-borne genes; green circles indicate presence of pMA02-borne genes. Percentages refer to the genes detected on plasmids of 18MA0850 and are indicated by shading.

Lastly, the pangenome analysis also showed that CDSs located on pMA01 and pMA02 were present in some of the external strains (Fig 9). Sixteen external strains harboured at least 31 ORFs (~70%) from pMA01. These isolates originated mostly from human (United Kingdom and USA), but also from animals, i.e., from swine and Cabot’s tragopan, and German dust. Particularly, in the genome assembly of strain MAV_061107_1842 from a patient in the USA (isolated in 2007), only a single pMA01 gene encoding an IS element was missing. Genes located on the smaller plasmid, pMA02, could only be found in a total of six human isolates, originating from the United Kingdom, USA and Australia. Except for the latter, which harboured 16 pMA02 genes, all CDSs were detected in these foreign isolates.

## Discussion

The *Mycobacterium avium* complex (MAC) comprises several species [3]. One of them, *M. avium,* is further divided into four subspecies, one of which is *M. avium* subsp. *hominissuis*, an opportunistic pathogen that is ubiquitous in the environment, e.g., soil, dust and water. It can also infect mammals, particularly humans and pigs. Also, other animals such as birds and cattle can be affected [13,54–56]. The isolates analysed in the current study originated from captive lowland tapirs and their enclosure environment. We aimed at determining the genomic similarity of these isolates assuming the occurrence of a micro-evolutionary event within strain 17MA0531 that differed in one MIRU-VNTR locus from the other collected isolates. In the past, epidemiological studies of mycobacterial populations relied on PCR-based typing approaches like MIRU-VNTR for assessing genetic diversity and epidemiological linkages [55,57–59]. However, it is known that this method does not accurately reflect phylogenetic relationships due to the influence of homoplasy [60]. By choosing WGS and *in silico* genotyping, the identity of the four German tapir-associated isolates was investigated in much greater detail.

The degree of genomic diversity varies between MAC species. While MAP genomes are highly stable, MAH exhibits a higher genomic diversity [5,60–64]. This high genomic diversity is reflected in significantly increased numbers of cgSNPs detected between MAH strains, i.e., three times higher for MAH than MAP and MAA/MAS [5,8,9,65]. The number of detected SNPs was also high in the genomes analysed within this study, although the strains originated from a single location. The phylogenetic network based on the SNP analysis and the PHI test indicated the presence of recombination events. By removing SNP sites influenced by recombination, the SNP differences decreased considerably from several thousands to 3-41 cgSNPs, values that would be expected for transmission events. *In vivo* studies showed that the annual mutation rate of MAH ranges between 5.25 and 6.22 SNPs [66,67], exceeding that of other MAC species by far (0.5-1 SNP/year) [5,60]. Particularly, isolates 17MA0531 and 18MA0854 from sputum and one of the tapirs, respectively, exhibited merely three SNPs. Considering the SNP accumulation rate, it can be assumed that the sputum isolate originated from tapir #2, as 18MA0854 was isolated one year later. It cannot be ruled out that Gubbins’ recombination filtering was too strict, removing also informative sites. Thus, additional genotyping methods were used for confirmation.

All genotyping methods applied in the current study gave coherent results regarding the genomic similarity of the strains, i.e., the sputum isolate and tapir #2 isolate were most similar, whereas 18MA0850, isolated from tapir #1, was the next closest and 17MA0524, isolated from the pool, differed the most from the other isolates. Remarkably, 17MA0531 was the only strain with a differing INMV pattern, but still was genomically the closest to one other of the three strains. Thus, this difference in the MIRU-VNTR profile can be considered a micro-evolutionary event that is not indicative for estimating the true genomic similarity to the strains. This underlines that VNTR-based results have to be interpreted with caution, as repeat regions are rapidly mutating.

Despite the high differences in SNPs, the genomic structure between the four strains was largely congruent. MAH is known for its high degree of genomic diversity with deletions and acquisition of genetic elements occurring at high rates compared to other mycobacterial species [65]. Genomic islands constituting strain-specific regions are widely distributed and highly variable among this subspecies [9,10,65,68]. Particularly, strain 17MA0524 harboured large strain-specific regions often associated with IS elements carrying genes predominantly involved in lipid transport and metabolism, which is a common trait of genomic islands in MAH [10,69].

Different approaches were used in this study for determining not only the genomic similarity of the four strains to each other but also to place them within the context of other MAH isolates. To date, seven MAH lineages have been defined [59] and it can be assumed that the four German isolates belonged to lineage SC4, as they clustered with OCU556, a member of SC4 [70], and showed lower ANI values to strains from other lineages. In contrast to lineage SC2, which is the closest to SC4, genomes of the latter exhibit high recombination rates resulting in a distinct mosaic genome structure. Particularly, SC4 genome regions were detected that originated from SC2 [70]. This was also observed for 17MA0524, that harboured a large genome region unique to this strain, even when compared to other genomes of MST ST9 and closely related STs. This region was nearly identical to a chromosomal region of MAH104, which is a member of the SC2 lineage. Originally isolated in 1983 from an AIDS patient in the USA, MAH104-like strains have been identified across numerous patients over the recent decades [71].

Such strain-specific elements are most likely acquired via horizontal gene transfer, which is very common among MAH, resulting in an open, highly diverse pan-genome of this subspecies [8,9,60,61,65]. This high variability results in a relatively low percentage of core genome genes (72%) in the pangenome analysis, even though predominantly MAH strains of MLST ST9 were included in this analysis. Many of these strains were found to be close relatives based on ANI analysis, with ANI values exceeding 99.4%, underlining the diversity of this subspecies. Owing to this high plasticity of MAH genomes, it was suggested that MAH actually comprised of several subspecies [8].

In accordance with the mosaic nature of SC4 lineage MAH, the r/m value calculated in the cgSNP analysis here was considerably high, meaning that recombination has an increased impact on diversification compared to base mutation. Interestingly, the removal of recombination-affected SNP sites did not impact the topology of the tree to a large extend which could indicate that the selective pressure is not high [72].

All of the here investigated isolates contained two plasmids, termed pMA01 and pMA02, that varied in size due to the integration of IS elements. Plasmids have been reported in many other MAH strains before, although these were usually larger, e.g., 78-194 kb [61,63,73]. Plasmid pMA01 was highly similar to the linear plasmid pMA100 from MAH strain 88Br. Unfortunately, no further information or whole genome sequence was available from that strain at the time of writing. However, pMA100 was markedly larger (116 kb). Also Dragset, Ioerger [63] found similarities of a 78 kb plasmid from MAH strain 11, originating from a HIV-positive patient, to pMA100.

Interestingly, pMA100 was shown to be transferrable to other mycobacterial species [73], which can be assumed for pMA01 and pMA02 as well, due to presence of relaxase-encoding genes. Genes encoded on pMA01 and pMA02 were also detected in some of the foreign strains included in the analysis and it can be expected that these strains contained derivatives of the plasmids. Evidently, derivatives of pMA100 are widespread among MAH, as MAH strain 11 belonged to a different MAH lineage (SC3) [70] than the strains studied here. However, no conclusion can be drawn on the plasmid transmission route. It was remarkable that in strains with the highest identity to 17MA0524 none of the two plasmids could be detected, which could indicate that 17MA0524 acquired the plasmid horizontally.

The question of the source of infection is of great concern in epidemiological studies. However, transmission routes of MAH often remain elusive [74,75] due to the ubiquitous distribution and high genetic variability of these bacteria. In Germany, reports of MAH isolation from dust, soil and birds exist [13,56,76]. In the previous study [11], the isolation of the four MAH strains investigated in this study was reported, from water, sputum and tapirs. Regarding the fact that these strains belong to the SC4 lineage which is predominant in central Europe, USA and Russia, it is most likely that the infection source is endemic, underlining the fact that environmental reservoirs might play an important role in contracting mycobacteriosis [57,58,77]. Regarding the genomic differences between the two tapir isolates, 18MA0850 and 18MA0854, it cannot be reliably concluded whether a transmission between the animals had occurred, whether it is a mixed infection from the same source or whether the sources of infection are different. Mixed MAH infections have been found previously in zoo animals as well as patients [5,67]. A major drawback is the lack of knowledge about MAH diversity in Europe but also from other continents and the resulting missing high-quality genome sequences in public databases. It can be assumed that the MAH diversity in Germany is high, as strains have been assigned to lineages SC2, SC3 and SC4 [70].

The current study illustrated the high genomic variability of MAH strains even in a limited spatial environment such as a zoo enclosure. Our results supported the findings by Yano, Iwamoto [9] and others that the phylogenetic network representing the genetic population structure of MAH is highly complex, suggesting an increased frequency of recombination between lineages. This variability might be one reason why MAH has the potential to infect a broader host spectrum than MAA/MAS or MAP. Additionally, it might enable MAH to adapt faster to adverse environmental conditions, among others also antibiotic therapy, which can make therapy more difficult, if not impossible.

## Supporting information

S1 File Supporting files include S1–S6 Tables and S1-S4 Figs and are accessible via Zenodo under https://doi.org/10.5281/zenodo.12918954.
(DOCX)
