# Supplementary material for: Comparative genomics of Mycobacterium avium subsp. hominissuis strains within a group of captive lowland tapirs
Source: PLoS One. 2025 Apr 1;20(4):e0320499. doi: 10.1371/journal.pone.0320499 (PMC11960956; doi:10.1371/journal.pone.0320499)
Supplement: S1 File — Supporting files include S1–S6 Tables and S1-S4 Figs and are accessible via Zenodo under https://doi.org/10.5281/zenodo.12918954. (DOCX) [file pone.0320499.s001.docx]

**Supporting Information for**

**Comparative genomics of *Mycobacterium avium* subsp. *hominissuis* strains within a group of captive lowland tapirs**

Hanka Brangsch^1^, Sandra Marcordes^2^, Anne Busch^3,4^, Michael Weber^5^, Silver A. Wolf^6^, Torsten Semmler^6^, Dirk Höper^7^, Sten Calvelage^7^, Jörg Linde^1^, Stefanie A. Barth^5^*

Supporting files include Tables S1-S6 and Figs S1-S4 and are accessible via Zenodo under doi: 10.5281/zenodo.12918954.

**S1 Table. Antimicrobial resistance genes predicted in silico.** Potential antimicrobial resistance genes detected in silico in four German MAH isolates using the CARD database. Besides the name of the gene, the potential resistance mechanism and according antibiotic is listed, as given in the CARD database. [PDF]

**S2 Table. Virulence-associated genes predicted in silico**. Virulence-associated genes predicted in silico in four MAH strains from Germany. As comparison, the virulence genes present in reference strain MAH 104 are given. The first two columns indicate the mechanism and the nomenclature of the genes. [CSV]

**S3 Table. Insertion elements predicted in silico.** Number of insertion elements per IS family predicted in silico in four German MAH strains. [CSV]

**S4 Table. Foreign strains used in this study**. Table of strains from repositories (GenBank, RefSeq, SRA) that were included in the analysis with their corresponding metadata and quality statistics. In the first column, the type of analysis that the strains were used for, is given. [CSV]

**S5 Table. Sequencing quality raw data.** Table showing the sequence quality values of the Nanopore and Illumina datasets as well as their analysis using BUSCO and conFindr. [CSV]

**S6 Table. Primers for in silico genotyping.** List of primers for in silico PCR usage. In silico PCR was conducted for species identification and allele-based typing of the MAH strains. [CSV]

**S1 Fig. Alignment of recF genes from four German MAH strains.** Identical bases are shaded in black, whereas variants are shaded white or grey. [CSV]

**S2 Fig. Minimum spanning tree based on cgMLST analysis.** Minimum spanning tree based on cgMLST analysis of German MAH strains and MAH genomes downloaded from NCBI and assembled from SRA data. The cgMLST scheme was designed in ad hoc fashion in this study. Clusters are shown by grey shading and were defined by a cut-off of 1150 alleles. Colours indicate MLST sequence types. Numbers on the branches give allele differences. [JPEG]

**S3 Fig. Tanglegrams comparing trees generated by different genotyping approaches**. Tanglegrams comparing A) maximum likelihood trees of SNP alignments before and after filtering for recombination, B) neighbour joining tree based on cgMLST allelic distances and maximum likelihood tree of cgSNP alignment before recombination filtering, C) neighbour joining tree based on cgMLST allelic distances and maximum likelihood tree of cgSNP alignment after recombination filtering by Gubbins. Strains that change their clustering between the two compared trees are coloured for better visibility. Lines between the trees connect identical isolates. [JPEG]

**S4 Fig.** **Pan genome analysis of MAH strains of MLST STs 9, 250 and 196**. Complete visualization of the pan genome analysis result including a maximum likelihood tree based on core gene alignment of strains of MLST STs 9, 250 and 196 meeting the quality criteria described in the methods section. The presence of a gene is indicated by blue colour. The leafs contain the accession numbers of the strains. [JPEG]
